# Evaluation of Antioxidant, Cytotoxic, Anti-Inflammatory, Antiarthritic, Thrombolytic, and Anthelmintic Activity of Methanol Extract of *Lepidagathis hyalina* Nees Root

**DOI:** 10.1155/2022/2515260

**Published:** 2022-02-15

**Authors:** Shafiqul Islam, Fowzul Islam Fahad, Arifa Sultana, Syed Al Jawad Sayem, Shawon Baran Roy, Mohammad Nazmul Islam, Arpita Roy, Mohammed Abu Sayeed

**Affiliations:** ^1^Department of Pharmacy, International Islamic University Chittagong, Chittagong-4318, Bangladesh; ^2^Department of Pharmacy, Faculty of Pharmacy, University of Dhaka, Dhaka-1000, Bangladesh; ^3^Department of Biotechnology, School of Engineering & Technology, Sharda University, Greater Noida, India

## Abstract

*Lepidagathis hyalina* Nees is an ethnomedicinally potential Asian herb, locally used to treat cardiovascular diseases and coughs. The study was intended to evaluate qualitative and quantitative investigation to ensure numerous pharmacological properties of methanol extracts of *L. hyalina* Ness root (MELHR). MELHR manifested strong radical scavenging activity in the reducing power and DPPH (1, 1-diphenyl-2-picrylhydrazyl) assays, and phenol and flavonoid in the quantitative assays. In the study of the thrombolytic assay, MELHR showed moderate explicit percentage of clot lysis (29.39 ± 1.40%) with moderate (135.35 *µ*g/mL) toxic properties. The *in vitro* anti-inflammatory activity was evaluated by the inhibition of hypotonicity-induced RBC hemolysis, whereas the plant extract exhibited a significant (p*p* ˂ 0.005) dose-dependent inhibition and the highest inhibition was found 55.01 ± 3.22% at 1000 µg/mL concentration. Moreover, the MELHR also showed significant (*p* < 0.005) dose-dependent potentiality on protein denaturation which is considered as antiarthritic activity, and the peak inhibition was found significant (71.97 ± 2.71%) at 1000 *µ*g/mL concentration. MELHR also exhibited the dose-dependent and statistically significant anthelmintic potential on aquarium worm (*Tubifex tubifex*). So, the present investigation suggests that *L. hyalina* could be the best choice for the management of cardiovascular, inflammation, arthritis, and anthelmintic diseases. Further investigation should be necessary to determine behind the mechanism of bioactivity and therapeutic potential of this plant.

## 1. Introduction

Free radicals are major concern for biological evolution and also have certain beneficial effects on some species [1]. An imbalance between the accumulation and production of ROS in tissues causes oxidative stress [2, 3]. Oxidative stress triggers inflammation, which in chronic conditions results in atherosclerosis formation, thrombosis, plaque rupture, myocardial injury, and failure like serious cardiac diseases [4, 5]. Different epidemiological studies express that some inflammatory mediators not only facilitate the proliferation of malignant cells in the microenvironment of the tumor, but also induce metastasis and angiogenesis and redefine the responses of hormones, chemotherapeutic agents, and overall adaptive immunity [6]. Some bioactive compounds such as capsaicin, catechins, lycopenes, cucurbitacin B, isoflavones, phenethyl isothiocyanate, benzyl isothiocyanate, and piperlongumine have been proved to exert effective pharmacological activities to treat cancer [7]. Certain phytochemicals like thymol, berberine, curcumin, lycopene, epigallocatechin, resveratrol, vanillin, and sulforaphane may also reduce the possibility of the onset of several types of cancer [8–11].

Arthritis becomes a common cause of disability in developed countries nowadays, which is attributed to pain, restricted joint movement, and synovial membrane inflammation [12, 13]. Fibroblast-like synoviocytes (FLSs) facilitate both the propagation of joint damage and inflammation by producing proinflammatory mediators like matrix metalloproteins (MMPs), interleukins (IL6, IL8), and prostaglandins (PGE2) [14]. For this reason, anti-inflammatory agents can also be used as a long-term treatment of rheumatoid arthritis [15]. Thrombolytic agents activate plasminogen to plasmin, which clears the fibrin mesh. As a result, the clot becomes soluble and blood flow gets normalized [16]. As the consequences of inflammation spread over a wide range of actions like asthma and atherosclerosis, multitargeted therapeutic approaches can be appeared prevailing over combination therapy or just single-target drugs [17, 18]. Besides, the antioxidant properties containing agents may prevent inflammation and thrombotic as well as help to prevent cardiovascular diseases [19].

Helminthiasis (worm infestation) is a macroparasitic ailment in which parasitic worms such as nematodes, trematodes, cestodes, and other parasitic worms infest the human and animal body and cause a variety of disorders like pneumonia, malnutrition, eosinophilia, anemia, etc. It exacerbates global economic and social difficulties, particularly in tropical areas [20]. According to the World Health Organization, helminthiasis affects 1.5 billion people or 24% of the world's population [21]. However, in developing countries, it has become a major public health issue owing to anthelmintic misuse, which has resulted in the development of anthelmintic resistance in parasitic worms [22].

However, antioxidants (e.g., arginine, glutathione, taurine, selenium, tea polyphenols, superoxide dismutase, and vitamin C, E, and A) overcome the oxidative stress by scavenging free radicals [2,3]. Unfortunately, plenty of synthetic agents used for the treatment of these diseases have potential side effects and also cannot afford adequate therapeutic significance [23, 24]. Therefore, the drugs, which can generate effect against parasitic worms, scavenge RONS, inhibit inflammation, exhibit thrombolytic activities, and give minimal side effect, would be a jackpot in the drug development process [25]. Plants have diversified phytochemicals obtained from secondary metabolism [26] that give diversified biological activities including some good pharmacological actions [27]. For instance, lichens, *Allium sativum* (garlic), and *Hydrastis canadensis* are good options of antimicrobial activities with lesser side effects than their synthetic counterparts like streptomycin and Aureomycin [28]. So, the popularity of traditional medicine is increasing in both developed and developing countries for its low cost and less side effects [29]. Additionally, incorporating plant-derived bioactive compounds in the conventional system is also increasing day by day.

*Lepidagathis hyalina*, also known as Curved Lepidagathis, is a wild herb from the *Acanthaceae* family that has various therapeutic potentials including antimicrobial and antidiabetic activities [30, 31]. This plant has been reported in various subtropical locations across the world, particularly in the Indian subcontinent. In Bangladesh, it is mostly found in hill tract regions which have a long history of use in the treatment of coughs and cardiovascular disorders [30, 32]. According to a prior study, a biological substance called triterpenoid saponin ((3-*β*-O-[*α*-L-rhamnopyranosyl (1⟶4)O-*β*-D-glucopyranosyl] 16-*α*-hydroxy-olean-12-en (13)-28-oic acid)) identified in the leaves of this plant has antibacterial and antifungal properties, and also the stem part of this plant manifested several pharmacological properties which are reported as antioxidant, thrombolytic, antidepressant, and anxiolytic activity [30, 32]. To verify its ethnomedicinal uses, we aim to investigate several *in vitro* tests on *Lepidagathis hyalina* Ness.

## 2. Methods and Materials

### 2.1. Chemicals

Sodium carbonate, ferric chloride, folin-ciocalteu reagent (FCR), trichloroacetic acid, methanol, potassium ferrocyanide, potassium acetate, aluminum chloride, hydrochloric acid, and sulfuric acid were purchased from Merck (Darmstadt, Germany). Diclofenac sodium and levamisole were brought from ACME Laboratories Ltd. (Dhaka, Bangladesh). Sodium acetate, quercetin, and 1, 1-diphenyl-2-picrylhydrazyl (DPPH) were collected from Sigma Chemical Co. (St. Louis, USA). Lyophilized streptokinase vial (1500000 IU) and vincristine sulfate (1 mg/vial) were gained from Beacon Pharmaceutical Ltd. (Dhaka, Bangladesh). Ultraviolet-Vis spectrophotometer (Shimadzu, Japan) was applied to take absorbance for this experiment. Specified reference-tagged chemicals were used in this research project which was an analytical grade reagent.

### 2.2. Collection

The roots of *Lepidagathis hyalina* Ness were collected as fresh condition from the Golden Temple Hill area, Bandarban, Chittagong, Bangladesh. After collection, the plant taxonomy was authenticated by Prof. Dr. Sheikh Bokhtear Uddin (Department of Botany, University Chittagong, Chittagong–4331, Bangladesh), and also identified and confirmed by Prof. Dr. Abu Sayeed (Department of Pharmacy, International Islamic University Chittagong, Chittagong-4318, Bangladesh).

The roots of *L. hyalina* were washed and stored in shade at low temperature for two weeks and after that ground into coarse powder through a blender machine. The blended powder of root was put in an impermeable container with a sufficient volume of methanol at room temperature for 10–14 days, and the mixed solution shook vigorously. A rotary evaporator was applied to filter the mixed solution at 40–50°C. By this process, a deep green sticky semisolid was formed and kept in a refrigerator until further study. All of the research design and protocols have been approved and authenticated by the P&D committee (Pharm-P&D17/08′'-19), Department of Pharmacy, International Islamic University Chittagong, Chittagong, Bangladesh.

### 2.3. Phytochemical Screening

MELHS was experimented for preliminary qualitative phytochemical analysis through the standard determination method of phytochemicals (e.g., quinones, carbohydrates, alkaloids, reducing sugar, polyphenols, phenols, flavonoids, resins, glycoside, phlobatannins, xanthoproteins, triterpenoids, coumarins, cardial glycoside, cholesterols, etc.) [33].

### 2.4. Antioxidant Activity Test

#### 2.4.1. DPPH Radical Scavenging Assay

Free radical scavenging assay of methanol extract of *L. hyalina* Ness roots (MELHR) was examined through the method described by Tayab et al. [34]. The DPPH reagent used all concentrations of MELHR and kept 30 mins in the darkened room. After incubation, the absorbance was recorded at 517 nm using a UV spectrophotometer against a blank solution. Ascorbic acid was subjected as a reference standard. The free radical scavenging assay was calculated by the equation as follows:

(1) $$\begin{array}{c} \%\text{ scavenging activity}=\frac{\text{Ac}-\mathrm{As}}{\text{Ac}}\times 100. \end{array}$$

Here, Ac  = absorbance of the control and As  = absorbance of the sample.

#### 2.4.2. Reducing Power Assay

The method of Sarkar et al. was conducted to evaluate the reducing power assay of MELHR [35]. 1 milliliter of several serial diluted concentrations (62.5 to 1000 *µ*g/mL) was made and then mixed with 2.5 ml of 0.2 M phosphate buffer (pH 6.6) and 1% potassium ferricyanide. At 50°C temperature, the mixed solution was incubated for reaction completion. About 2.5 ml of 10% trichloroacetic acid was added and centrifuged the mixer for 10 mins at 3000 rpm. The formed supernatant solution was dispelled after centrifuging period. Then, a half milliliter of 0.1% ferric chloride and 2.5 milliliter distilled water were summed up and then investigated the absorbance at 700 nm on a UV spectrophotometer. The blank and reference standard that applied in this experiment was phosphate buffer and ascorbic acid, respectively.

#### 2.4.3. Total Phenolic Content Test

The total phenolic content of MELHR was measured by the following method, where gallic acid is used as standard [36]. 1 ml of extract solution and 1 ml of the standard solution containing different concentrations were taken to the different test tubes. 5 ml diluted FCR and 5 ml solution containing different concentrations of 7.5% sodium carbonate were added to each test tube one after another. The test tubes were incubated at 25°C for 20 minutes to facilitate the reaction. The test tubes and a blank sample were placed in the UV machine, and absorbance was taken to 760 nm. A standard curve was generated based on the gallic acid, and total phenolic content (TPC) was calculated.

#### 2.4.4. Total Flavonoid Content Test

The content of total flavonoid content of MELHR was measured by aluminum chloride colorimetric method using quercetin as standard [32]. 1 mL of extract solution and 1 ml of standard solution containing different concentrations were taken to the different test tubes. 3 ml methanol, 200 *μ*l aluminum chloride (10%), 200 *μ*l of 1 M potassium acetate, and 5.6 milliliters of distilled water were added to each test tube one after another. The final mixture was placed in incubation for 30 minutes to facilitate the reaction. Then, the sample, standard, and a blank were placed into UV machine, and absorbance was measured at 420 nm. Total flavonoid content was shown as mg of quercetin equivalent/gm of dried extract.

### 2.5. Brine Shrimp Lethality Bioassay

The cytotoxic properties of MELHR were investigated by the method of Alam et al. with slight modification [37]. Artemia salina leaches (brine shrimp eggs) were subjected as test organisms to evaluate the toxic potential. To develop artificial seawater, the seawater (38 g/L) and 1N NaOH were mixed well and adjust the pH 8.5. After that, shrimp eggs were hatched in this mixer and placed at room temperature under a constant oxygen supply. Around 2 days were allowed for maturing the shrimp eggs into larvae which were named nauplii. The crude extract was dissolved in DMSO (5 mg/mL) with artificial seawater and considered as a test sample which was led to serial dilution and obtained several concentrations (31.25 to 1000 *µ*/mL). Vincristine sulfate was used in this experiment as a positive control as the preceding method in serial concentrations between 0.125 *µ*g/mL to 10 *µ*g/mL. In each experimental vial, ten alive nauplii were added and incubated at room temperature under the light. After incubation, an amplifying glass was applied to calculate the living nauplii in each vial and record the number. The mortality percentage of nauplii was figured out according to the equation:

(2) $$\begin{array}{c} \text{percentage }\left(\text{\%}\right)\text{of mortality }=\;\frac{N_{0}-N_{1}}{N_{0}}\times 100, \end{array}$$

where *N*_0_ = number of nauplii taken and *N*_1_ = number of nauplii alive.

### 2.6. Anti-Inflammatory Assessment

#### 2.6.1. Erythrocyte Suspension Preparation

Alsever solution (0.5% citric acid, 0.8% sodium citrate, 2% dextrose, and 0.42% sodium chloride) was taken and equally mixed with fresh whole blood that was taken from some healthy volunteers. Then, the blood solution was centrifuged at 3000 g for 10 mins. After that, isosaline was subjected to wash the packed cells, then made 10% v/v solution, and kept at 4°C before use in this experiment.

#### 2.6.2. Hypotonic-Induced Human Red Blood Cell Hemolysis

The anti-inflammatory effects of MELHR were inspected on hemolysis of human red blood cells (HRBCs) induced by a hypotonic solution and were evaluated using the described method with slight modification [18]. Different concentrations (31.25 to 1000 *µ*g/mL) of crude extract and diclofenac sodium were taken, and then, add 0.5 mL of stock erythrocyte (RBC) suspension, 1 mL of 10 mM sodium phosphate buffer (pH 7.4), and 2 mL of hypotonic solution (50 mM sodium chloride), respectively. 0.5 mL of stock erythrocyte (RBC) suspension and the hypotonic-buffered solution were mixed and considered as a control sample solution. The different mixer that was made in this experiment was incubated for half an hour at 37°C temperature and then centrifuged for 20 mins at 3000 g. After centrifugation, the supernatant solution was subjected to calculate the absorbance at 560 nm on a UV spectrophotometer. The percentage of inhibition was counted through the equation as follows:

(3) $$\begin{array}{c} \text{percentage }\left(\text{\%}\right)\text{of inhibition}=\frac{{\text{OD}}_{1}-{\text{OD}}_{2}}{{\text{OD}}_{1}}\times 100, \end{array}$$

where OD_1_ = optical density of the hypotonic-buffered saline solution and OD_2_ = optical density of test sample in a hypotonic solution.

### 2.7. Antiarthritic Assay

The antiarthritic activity of MELHR was investigated by the method of the protein denaturation technique [18]. The reaction mixture of test solution (0.5 mL) consisted of 5% w/v aqueous solution of bovine albumin (0.45 mL) and test sample (0.05 mL), and the control solution (0.5 mL) comprised a mixture of 0.45 mL bovine serum albumin (5% of w/v aqueous solution) and 0.05 mL of distilled water. Different concentrations (31.25 to 1000 *µ*g/mL) of MELHR and diclofenac sodium were taken. 1N HCl was applied to adjust the pH 6.3 of the solutions and then incubated first for 20 mins at 37°C temperature. After that, all solutions were kept in an incubator for half an hour again at 57°C. After the incubation process, all the solutions were cooled, and then add 2.5 milliliters of phosphate buffer. The UV-visible spectrophotometer was subjected to measure the absorbance of the solutions at 416 nm. The control manifests 100% protein denaturation. The following equation was used to measure the percentage inhibition of protein denaturation:

(4) $$\begin{array}{c} \text{percentage }\left(\text{\%}\right)\text{of inhibition}=\frac{A_{C}-A_{S}}{A_{C}}\times 100, \end{array}$$

where *A*_*C*_ = absorbance of the control and *A*_*S*_ = absorbance of the sample.

### 2.8. Thrombolytic Activity

The *in vitro* thrombolytic assay was performed using streptokinase vial in the same method described by Hasnat et al. [38]. 0.5 ml of venous blood withdrawn from healthy volunteers was placed in ten sterile microcentrifuge tubes which were previously weighed. Each tube was placed in incubation for 45 min at 37°C or clot formation. When the clot formation is completed, serum was gently removed from each test tube without disrupting the clot. 100 *µ*l of (10 mg/ml) methanol extract was added to the preweighed clot. 100 *µ*l streptokinase was added to the positive control group and 100 *µ*l of distilled water in the negative control group. All the tubes were further incubated for 90 min at 37°C for clot lysis. After removing the fluid, the weight of the clot was further measured and the difference in weight was calculated. The percentage clot lysis was computed using the formula:

(5) $$\begin{array}{c} \text{\% clot lysis }=\left(\frac{\text{weight of clot after removing the fluid}}{\text{weight of clot}}\right)\times 100\text{.} \end{array}$$

### 2.9. Anthelmintic Activity

The anthelmintic activity of crude extracts was measured by the method described by Ajaiyeoba et al. with some minor modifications [39]. In this experiment, the aquarium worm *Tubifex tubifex* was subjected to find out anthelmintic potential because it has anatomical similarity and belongs to the same group of intestinal worms. The sludge worm used in this experiment was collected from the aquarium shop of Chittagong. The experiment was divided into several groups; whereas the negative control group consists of only distilled water, the positive control group consisted of the standard drug levamisole (1 mg/mL), and the test group consisted of different concentrations (5, 8 and 10 mg/mL) of crude extracts, respectively. In this investigation, 10 to 12 worms were placed in each Petri dish in five different groups. Then, 3 mL of the different concentrations of all groups was added to the Petri dish. The initial time, paralysis time, and the death time of the worms were observed and recorded carefully, in which paralysis time and death time of worms were considered as the evaluation of the anthelmintic activity of this experiment. When the worm's movement could not be observed after shaking vigorously, the paralysis time and the death time considered by the confirmation of worm's movement could not be observed either after shaking or when dipped in slightly warm water.

### 2.10. Statistical Analysis

GraphPad Prism version 7.00 (GraphPad Software Inc., San Diego, CA) was used to analyze the experimental results. The data were presented as mean ± SEM (standard error mean), in which ^*∗*^*p* < 0.05, ^*∗∗*^*p* < 0.01, and ^*∗∗∗*^*p* < 0.001 were considered as statistically significant. The one-way ANOVA was measured by following Dunnett's test compared to the negative control. All the assays were conducted as triplicate and repeated three times each for the consistency of the result and statistical function.

## 3. Results

### 3.1. Phytochemical Screening

The qualitative phytochemical experiment of methanolic extracts of *L. hyalina* Nees root (MELHR) manifested the presence of carbohydrates, quinones, alkaloids, reducing sugar, phenols, polyphenols, flavonoids, triterpenoids, coumarins, and cardial glycosides. The yield of this experiment is summarized in Table 1.

### 3.2. Antioxidant Effect

#### 3.2.1. DPPH Radical Scavenging Assay

**Figure 1:**
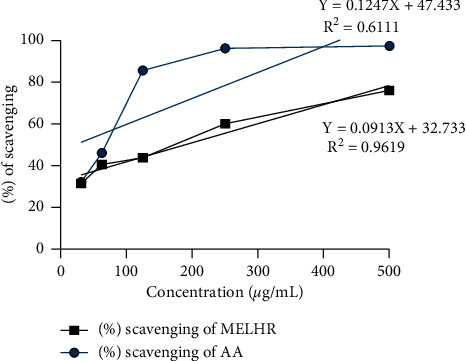
Percentage of radical scavenging activity by the DPPH (1, 1-diphenyl-2-picrylhydrazyl) assay of the MELHR (methanolic extract of *L. hyalina* Ness root) and standard drug ascorbic acid (AA) at differ concentrations.

The antioxidant activity of MELHR was investigated by DPPH free radical scavenging. The crude extract manifests potential antioxidant properties which were presented in Figure 1. The maximum antioxidant potency (76.18%) of MELHR has shown at 500 *µ*g/ml concentration, while standard ascorbic acid demonstrates a 97.49% effect at the same concentration. Here, the potentiality of scavenging properties was increased compared with the increased concentration. The IC_50_ values of MELHR and ascorbic acid were 189.01% and 20.59%, respectively, which was estimated via linear regression formula.

#### 3.2.2. Reducing Power Activity

**Figure 2:**
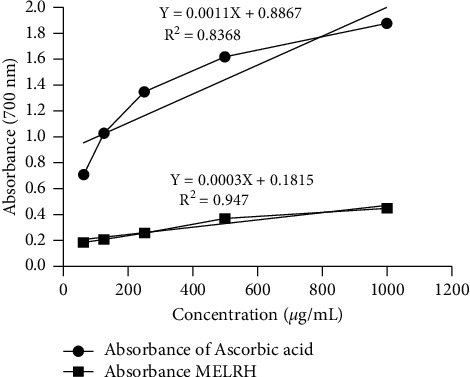
Reducing power of MELHR and standard drug ascorbic acid (AA) at different concentrations.

Reducing power is related to antioxidant properties in which the components containing reducing power can decrease the oxidized intermediated lipid peroxidation process. The reducing power properties of MELHR and ascorbic acid are summarized in Figure 2. We can see that both extract and standard displayed increased absorbance with the increased concentrations. The peak absorbance of MELHR was found 0.451 at 1000 *µ*g/ml concentration, whereas the standard has shown 1.88 absorbance at the same concentrations.

#### 3.2.3. Total Flavonoid and Phenol Contents

The total flavonoid and phenol content of the crude extract was estimated quantitatively. The result is displayed in Table 2. The flavonoid and phenolic potentials of MELHR were found at 41.40 ± 0.204 mg QE/gm and 98.61 ± 0.064 mg GAE/gm, respectively. Here, MELHR was carried out through linear regression equation (for flavonoid activity, equation stands as *y* = 0.0102*x *−* *0.0637; for phenol assay, it was *y* = 0.0039*x* + 0.033).

### 3.3. Cytotoxic Activity

**Figure 3:**
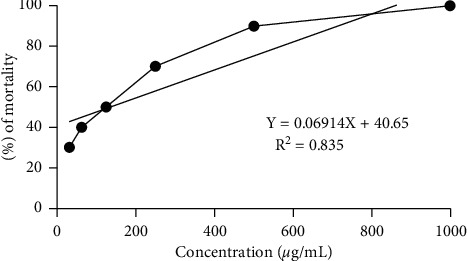
Percentage of mortality of brine shrimp at different concentrations of methanolic extract of *L. hyalina* Nees (MELHR).

The potential of cytotoxicity of crude extract was evaluated via brine shrimp cytotoxic assay. The fatality result of plant extract was assessed in Figure 3. Here, the LC_50_ value of MELHR was 135.35 *µ*g/mL which was demonstrated via linear regression equation (*y* = 0.0691*x* + 40.647) and the average percentage of mortality was 63.33%.

### 3.4. Anti-Inflammatory Effect

**Figure 4:**
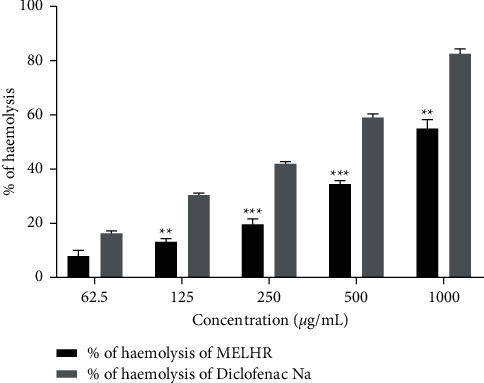
In vitro anti-inflammatory activity (membrane stabilizing assay) of methanolic extract of *L. hyalina* Ness root (MELHR). Values are expressed as mean ± SEM (*n* = 3); ^*∗*^*p* < 0.05 is statistically significant comparison with diclofenac Na followed by Dunnett's test.

The *in vitro* anti-inflammatory potential of MELHR is assessed in Figure 4, in which crude extracts manifest dose-dependently and significantly (*p *˂* *0.05) increased the anti-inflammatory properties. The peak percentage of inhibition of hemolysis by hypotonic solution and heat-induced hemolysis of crude extract was found 55.01 ± 3.22 at 1000 *µ*g/mL concentration, whereas diclofenac Na manifested 82.46 ± 1.92 at the same concentration.

### 3.5. Antiarthritic Effect

**Figure 5:**
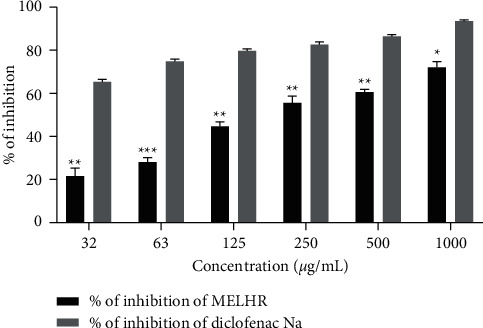
I*n vitro* antiarthritic activity (inhibition of protein denaturation assay) of methanolic extract of *L. hyalina* Ness root (MELHR). Values are presented as mean ± SEM; one-way analysis of variance (ANOVA) followed by Dunnett's test. ^*∗∗*^*p* < 0.01 and ^*∗∗∗*^*p* < 0.001 are considered as significant compared with the diclofenac Na.

The antiarthritic activity of MELHR on protein denaturation is presented in Figure 5. The plant extract showed dose-dependent inhibitory potency when compared to diclofenac sodium. The percentage of inhibition was 21.71 ± 3.53, 28.15 ± 1.88, 44.58 ± 2.03, 55.41 ± 3.25, 60.64 ± 1.22, and 71.97 ± 2.71 for MELHR; and 65.3 ± 1.09, 74.87 ± 0.89, 79.73 ± 0.82, 82.59 ± 1.4, 86.44 ± 0.73, and 93.59 ± 0.22 for diclofenac sodium at the concentration of 31.25, 62.5, 125, 250, 500, and 1000 *µ*g/mL, respectively.

### 3.6. Thrombolytic Effect

**Figure 6:**
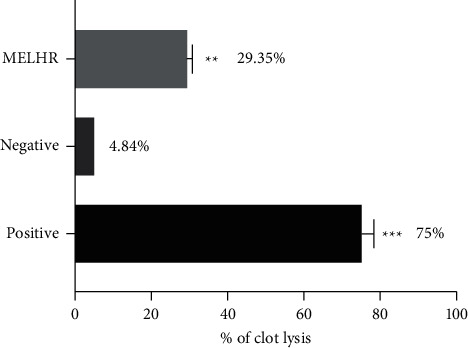
*In vitro* thrombolytic activity of MELHR. Values are presented as mean ± SEM; one-way analysis of variance (ANOVA) followed by Dunnett's test. ^*∗*^*p* < 0.05, ^*∗∗*^*p* < 0.01, and ^*∗∗∗*^*p* < 0.001 are considered as significant compared with control.

The thrombolytic property of MELHR is shown in Figure 6. The experimental record uncovers that MELHR has significantly (*p* ˂ 0.005) moderate (29.39 ± 1.40%) clot lysis properties compared with both positive and negative controls.

### 3.7. Anthelmintic Effect

The anthelmintic activity of MELHR was investigated on *Tubifex tubifex* worms which finding summarized in Table 3. In this investigation, at the 5, 8, 10 *µ*g/mL concentrations the extract manifested significant paralysis time 13.2 ± 0.842, 7.37 ± 0.684, and 4.5 ± 065 min; and death time 36.16 ± 3.096, 27.3 ± 2.197, and 15.53 ± 1.88 min, respectively, whereas the standard drug levamisole showed paralysis and death time 3.17 ± 0.189 and 6.5 ± 0.384 min, respectively, at 1 *µ*g/mL. The result indicated that the effect of anthelmintic was directly proportional to the concentrations of crude extract.

## 4. Discussion

Despite the availability of modern medicines, medicinal plants in developing countries are becoming increasingly popular as primary healthcare products because of their low cost and low side effects and toxicity [40, 41]. In this study, we conducted various tests to assess different health benefits like antioxidant, anti-inflammatory, antiarthritic, thrombolytic, cytotoxic, and antianthelmintic of the methanolic extract of *Lepidagathis hyalina* roots. Phytochemical screening of the methanolic extract of *L. hyalina* roots confirmed the presence of a wide range of chemical entities such as carbohydrates, quinones, alkaloids, reducing sugar, phenols, polyphenols, flavonoids, triterpenoids, coumarins, and cardial glycoside that can be associated with several pharmacological activities given by the plant extract.

The numerous artificial antioxidants have been replaced with plant-based polyphenols and flavonoids in recent years. Nature source antioxidants are obviously safer to consume than artificial antioxidants, which are suspected of harmful effects on health [8, 42]. The extracts of the plant were compared with conventional antioxidant ascorbic acid, which exhibited a dose-dependent antioxidant activity in DPPH testing. The IC_50_ values of crude extract were found 189.01%, which by comparison with ascorbic acid stand as 20.59%. Then, the connection of methanol extracts between TPC, TFC, reducing power, and DPPH was analyzed, indicating that MELHR has strong antioxidant potentiality [19]. However, the brine shrimp lethality test is a common technique to test cytotoxic action along with ion channel interference, enzyme inhibition, and antibacterial activity [43]. LC_50_ at low concentration with a quick response indicates that the plant extract is quite potent to give cytotoxic activity [16].

Since the membrane of erythrocytes is regarded to be analogous to the lysosomal membrane, the erythrocyte membrane stabilization test is considered to be an efficient tool for the anti-inflammatory drug screening process. The extracellular release of lysosomal contents by activated neutrophils is an important phenomenon in the pathophysiology of inflammation that is why membrane stabilization tests play a crucial role to assess inflammation [44, 45]. Both MELHR and diclofenac Na treatment groups significantly increased the percentage of hemolysis in a dose-dependent manner. Thus, the plant extract exhibited an anti-inflammatory effect in medium to high doses. One of the most well-known origins of arthritic diseases is the denaturation of tissue proteins. Denaturation of proteins can result in the production of autoantigens in some arthritic disorders [46, 47]. It might be caused by changes in electrostatic, hydrophobic, disulphide, and hydrogen bonds in proteins [47]. Here, the *in vitro* antiarthritic activity of MELHR was summarized in terms of inhibition of the protein denaturation method. The study demonstrated that both the standard drug and the plant extract increase inhibition level gradually with the increase in doses, but diclofenac Na was observed to give better results than plant extract indicating a better antiarthritic effect.

Thrombosis occurs as a result of hypercoagulation of the blood, damage to blood vessels, and blockage of blood flow within blood vessels. It is a life-threatening vascular complication of arthritis, myocardial infarction, pulmonary embolism, cerebrovascular ischemia, stroke, and venous embolism. Furthermore, venous thrombosis has been identified as the second greatest cause of cancer-related mortality [48]. The plant extract significantly (*p* < 0.05) reduced the percentage of clot lysis (29.35%) as moderate scale compared with standard. A significant difference between the percentage values of clot lysis of positive and treatment groups indicates the efficiency of plant extract to exert effective thrombolytic activity. In a previous study, carbon tetrachloride extract and dichloromethane soluble extract have shown an almost similar level of activity (24.89 ± 0.23 and 23.59 ± 1.29%, respectively) against streptokinase standard [49].

As part of a study to assess anthelmintic action, it was discovered that the crude extract causes dose-dependent paralysis, ranging from loss of mobility to death. The plant extract's paralysis and death time were compared to that of the standard medication levamisole. The anthelmintic activity of the test extract steadily increased, although at a higher concentration than levamisole. The fact that the plant extract includes a combination of components, but levamisole is a single component utilized as a medicine, might be the explanation. It was obtained from the previous studies that alkaloid, phenol, tannin, and terpenoids play an important role to give anthelmintic activity [50–52].

## 5. Conclusions

The complied pharmacological results indicated that this plant has a strong potentiality for the treatment of several diseases like arthritis, inflammation, cancer, etc. In our investigation, MELHR has been proved to have promising radical scavenging properties. Additionally, MELHR possesses significant arthritis, anti-inflammatory, thrombolytic, and anthelmintic activity with moderate toxic effects. This potentiality of MELHR could be due to the several bioactive compounds that are found in phytochemical analysis. Overall, *L. hyalina* Nees could be considered as a potential source for discovering the secondary metabolites which could be used for numerous pharmacological applications, and further studies are required to reveal the mechanism behind its potentiality.

## Figures and Tables

**Table 1:** Result of phytochemical screening of methanolic extract of *L. hyalina* Nees roots (MELHR).

Phytochemicals	Test types	Appearance	Results
Carbohydrates	Molisch's test	Reddish color ring form	++
Quinones	HCl test	Yellow color present	+
Alkaloids	Wagner test Mayer's test	A reddish-brown color Yellow color	++ +
Reducing sugar	Benedict's test Fehling's test:	Reddish color precipitate form Red precipitate form	++ ++
Phenols	FeCl_3_ test	Violet color form	++
Polyphenols	Ferric cyanide test	Blue-green color form	+
Flavonoids	Lead acetate test	Fluorescence yellow color form	++
Resins	FeCl3 test	No precipitation	−
Glycosides	Shinoda test	No deep red color	−
Phlobatannins	HCl test	No reddish precipitate form	−
Xanthoproteins	Xanthoprotein test	No reddish-brown precipitate form	−
Triterpenoids	Salkowski's test	Reddish-brown color form	+
Coumarins	Ammonia test	Green color form	++
Cardial glycosides	Legal test	Brown color	+
Cholesterols	General test	No red rose color	−

++: Highly present; +: moderately present; −: absent.

**Table 2:** Total phenol and flavonoid contents of methanol extract of *Lepidagathis hyalina* Ness root (MELHR).

Tested extract	Total phenol content (mg GAE/g dried extract)	Total flavonoid content (mg QE/g dried extract)
MELHR	98.61 ± 0.064	41.40 ± 0.204

MELHR: methanolic extract of *L. hyalina* Ness root.

**Table 3:** Anthelmintic activity of methanolic extract of *Lepidagathis hyalina* Ness root (MELHR).

Treatment and concentration	Time taken for paralysis in min	Time taken for death in min
Levamisole (1 *µ*g/mL))	3.17 ± 0.189	6.5 ± 0.384
MELHR (5 *µ*g/mL)	13.2 ± 0.842^*∗∗∗*^	36.16 ± 3.096^*∗∗∗*^
MELHR (8 *µ*g/mL)	7.37 ± 0.684^*∗∗*^	27.3 ± 2.197^*∗∗∗*^
MELHR (10 *µ*g/mL)	4.5 ± 0.65	15.53 ± 1.88^*∗*^

MELHR: methanolic extract of *L. hyalina* Ness root.

## Data Availability

The data used to support the findings of this study are included within the article.

## Abbreviations

MELHR: Methanol extract of *Lepidagathis hyalina* Nees root
BSA: Bovine serum albumin
DPPH: 1, 1-Diphenyl-2-picrylhydrazyl
FCR: Folin-ciocalteu reagent
SEM: Standard error mean
TPC: Total phenol content
TFC: Total flavonoid content
UV: Ultraviolet.
